# Dynamics of Rayleigh Fission Processes in ∼100 nm Charged Aqueous Nanodrops

**DOI:** 10.1021/acscentsci.3c00323

**Published:** 2023-05-31

**Authors:** Emeline Hanozin, Conner C. Harper, Matthew S. McPartlan, Evan R. Williams

**Affiliations:** Department of Chemistry, University of California, Berkeley, California 94720, United States

## Abstract

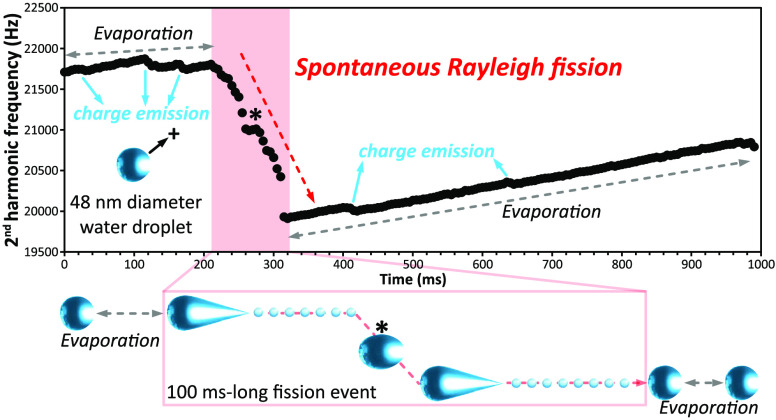

Fission of micron-size charged droplets has been observed using optical methods, but little is known about fission dynamics and breakup of smaller nanosize droplets that are important in a variety of natural and industrial processes. Here, spontaneous fission of individual aqueous nanodrops formed by electrospray is investigated using charge detection mass spectrometry. Fission processes ranging from formation of just two progeny droplets in 2 ms to production of dozens of progeny droplets over 100+ ms are observed for nanodrops that are charged above the Rayleigh limit. These results indicate that Rayleigh fission is a continuum of processes that produce progeny droplets that vary widely in charge, mass, and number.

## Abstract 2

Charge detection mass spectrometry reveals that the spontaneous fission of charged aqueous nanodrops occurs via many pathways that produce different numbers of progeny droplets over 2−100+ ms.

## Introduction

Charged droplets are formed by many natural processes, including mechanical breakup of water in ocean surf or discharges in thunder clouds,^1^ and they also play important roles in various technological applications^2^ and industrial processes.^3^ The breakup of charged droplets, originally described by Lord Rayleigh in 1882, occurs when the electrostatic repulsive force overcomes the cohesive force of surface tension.^4^ When the charge on a spherical droplet approaches or exceeds the Rayleigh limit, *q*_*R*_, instability arises leading to a fission event in which one or more smaller charged progeny droplets are produced (eq 1):
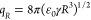
1where *R* is the droplet radius, γ is the surface tension, and ε_0_ is the permittivity of the surrounding media. Droplet fission has been investigated using a variety of methods,^5^ ranging from an early capacitor-type apparatus^6^ and Millikan condensers^7−9^ to more sophisticated electrodynamic ion traps^10^ and phase Doppler interferometry.^11^ However, the use of light scattering^5,12^ and/or high-speed camera^13^ detection methods has limited prior investigations to droplets with diameters above 4 μm.^5^ Varying results from many different solvents have been reported making comparisons and a general description of the Rayleigh fission process challenging.^14^ Generally, a single discrete fission event has been reported for droplets that are charged between 60% and 120% of the Rayleigh limit.^11,15^ After fission, the charge on the original droplet is reduced by 10% to 40%, while the mass decreases by just a few percent.^16^

Significantly less is known about the characteristics of progeny droplets that are produced by fission.^14^ Most experiments measure the properties of the initial droplet before and after fission so that information about the charge, the mass, and the number of progeny droplets are inferred or modeled.^7,13,15−19^ Estimates of the number of progeny droplets produced vary widely, typically <10^7,16,18^ but up to ∼100 has been reported.^13^ An especially elegant study by Duft et al. shows the breakup of 48 μm diameter droplets of ethylene glycol that resulted in formation of ∼100 small progeny droplets that carried away 33% of the charge on the original droplet and about 0.3% of its mass.^13,20^ This is perhaps the most detailed information about droplet fission that has been reported, with both the original droplet and the progeny droplets optically imaged. In these experiments, the large time-dependent electrical potential used to trap the droplets induces quadrupolar shape oscillations that lead to Coulomb instability and fission.^21^ These field-induced fission processes may not be descriptive of spontaneous fission of charged droplets.

Understanding spontaneous fission processes that occur in aqueous droplets is important in many applications, including electrospray ionization (ESI), a method that is used in thousands of laboratories worldwide to form gaseous ions from a wide range of molecules and molecular complexes for analysis by mass spectrometry (MS). Nanoelectrospray, commonly used in native MS applications, produces charged aqueous droplets with diameters that are initially on the order of a few 100 nm or less.^22^ Molecular dynamics simulations of highly charged water nanodrops with up to 23,000 water molecules (∼11 nm diameter)^23−27^ indicate that small minimally solvated ions, including atomic ions as well as peptides and proteins, can be ejected via formation of a thin liquid filament.^26,27^ Simulations also indicate that nanodrops containing highly charged ions can significantly distort and adopt star-shape morphologies before emitting charge via multiple jets.^25^ Experimentally, ejection of singly charged ions that carry away few solvent molecules was observed for droplets with diameters ≤32 nm.^28^ However, there have been no experimental observations for spontaneous charge-induced breakup of aqueous droplets between ∼40 nm and 1 μm, a range of droplet size important to native MS and many other phenomena. Moreover, limited information about the dynamics of the fission process has been reported, with fission generally occurring faster than the time scale of most prior experiments.^11,16,19,20,29^ Here, the first experimental observations of fission processes and dynamics of charged aqueous nanodrops with diameters between 40 and 120 nm are reported.

## Results and Discussion

### Water Evaporation from Aqueous Nanodrops and Fission

Positively charged nanodrops composed of water purified at a resistivity of 18.2 MΩ·cm and formed by ESI were trapped in an electrostatic ion trap of a charge detection mass spectrometer. The mass-to-charge ratio (*m*/*z*) is determined from the frequency of motion (*f*) and a trap-specific function of charged nanodrop energy (*C*(*E*)) shown in eq 2:
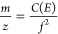
2

**Figure 1:**
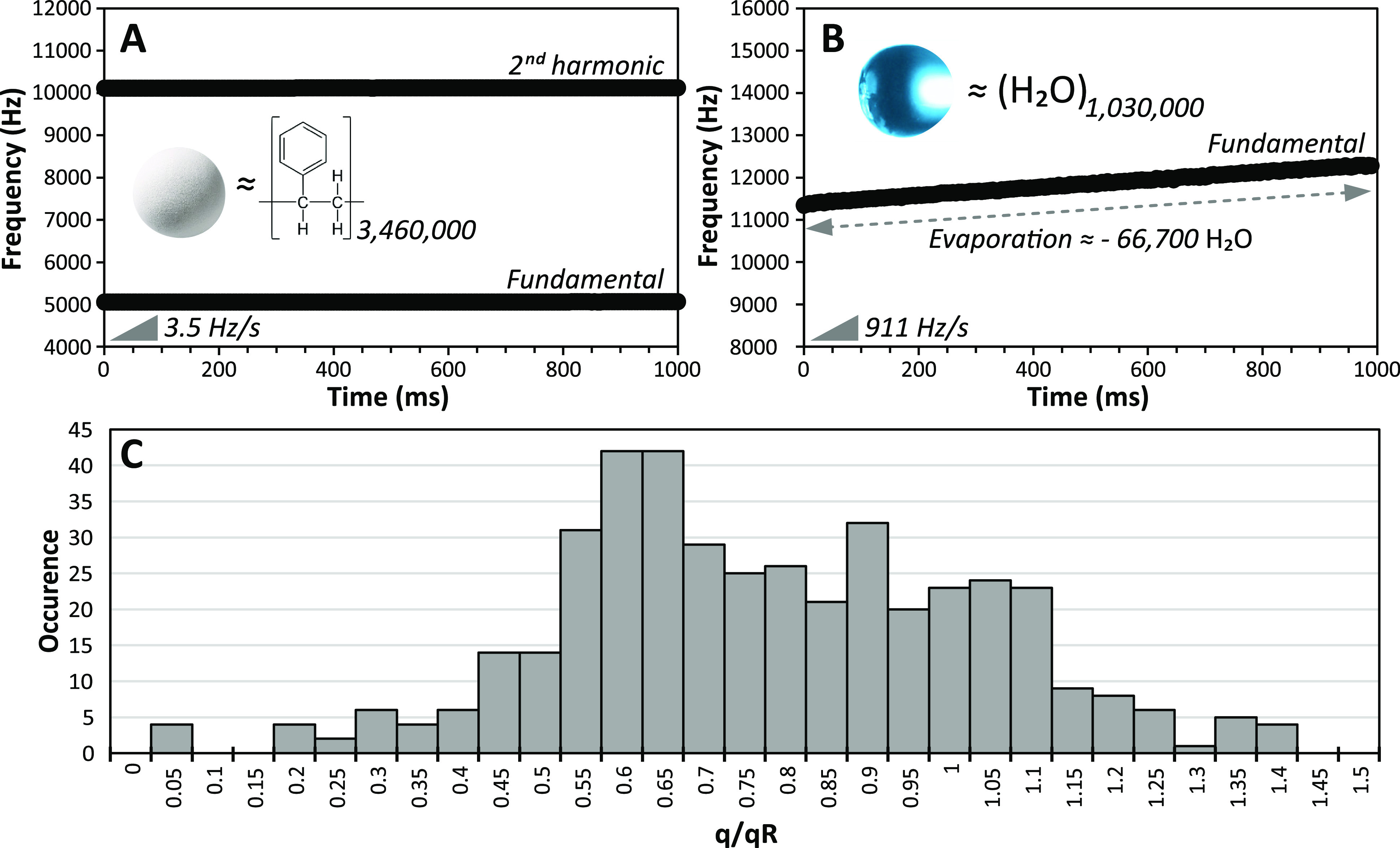
Frequency evolution of a dry nanoparticle, a water nanodrop and distribution of nanodrops charging relative to the Rayleigh limit. (A) Frequency evolution of a dry 103 nm diameter polystyrene nanosphere. (B) frequency evolution of a 39 nm diameter aqueous nanodrop undergoing evaporation. (C) Distribution of the charge relative to the Rayleigh limit (*q*/*q*_*R*_) of 425 water nanodrops undergoing exclusively evaporation during 0.5 s. The Rayleigh limit was calculated using the surface tension and density of water at ∼0 °C, 0.07564 J·m^–2^ and 0.9998 g·cm^–3^, respectively.^33^

Charge is obtained from the signal amplitudes of the fundamental and harmonic frequency. The frequency, signal amplitudes, and energy (*E*) of each charged nanodrop were continuously measured throughout the trapping period, making it possible to track the time evolution of both the charge (*z*) and the mass (*m*).^30−32^ For illustration, the measured fundamental frequency of motion of a dry 103 nm diameter polystyrene nanosphere (360.3 ± 0.8 MDa; 1178 ± 2 charges) is shown in Figure 1A. This frequency gradually increases by +3.5 Hz/s over a 1 s trap time. During the analysis, the mass of the ion remains the same, but the ion energy is continuously reduced by collisions with background gases, which leads to this shift in frequency. Under less energetic sampling conditions, positively charged aqueous nanodrops can be transmitted and trapped. The frequency of a 39 nm diameter aqueous nanodrop (18.0 ± 0.6 MDa; 359 ± 10 charges) increases continuously by 911 Hz/s (Figure 1B). The significantly higher shift in frequency compared to a dry polystyrene nanosphere is due to water evaporation that reduces both the mass and the energy of the nanodrop during the analysis. This frequency change corresponds to a mass loss of 1.2 MDa, corresponding to ∼66,700 water molecules evaporating from the nanodrop. Only a few thousand water molecules are lost for ≤32 nm aqueous nanodrops over the course of a 1 s trap time,^28^ consistent with their smaller size.

For 458 positively charged water nanodrops that were trapped for at least 0.5 s and up to 1 s, 425 only undergo water evaporation. The charge of these nanodrops relative to the Rayleigh limit (*q*/*q*_*R*_) is shown in Figure 1C. These nanodrops are charged between ∼5% and ∼140% of *q*_*R*_, but the majority of nanodrops are charged between 55% and 110%. The significant drop-off above 110% may be due to fission events that occurred prior to the electrostatic ion trap that would be expected to lower the charge of the resulting nanodrop by ∼30–40%, consistent with a significant distribution of nanodrops centered ∼60%. The Rayleigh limit was calculated using the surface tension and density of water at ∼0 °C, 0.07564 J·m^–2^ and 0.9998 g·cm^–3^, respectively.^33^ Although the temperature of these nanodrops is lower because of evaporative cooling, it appears that this value of surface tension indicates a size range of nonfissioning nanodrops that is reasonable, but further investigation is required.

Charged aqueous nanodrops can undergo fission while they are trapped. Out of the 458 nanodrops, 33 undergo fission during the trapping period. Seven of these nanodrops are described in significant detail below. These nanodrops were selected because they are representative of the broad range of fission events that were observed. All nanodrops that exhibit a discharge event are charged well above the Rayleigh limit, ranging from 123% and 172% of *q*_*R*_. If the interfacial tension and density of ice water at ∼ −38 °C (0.09 J·m^–2^ and 0.917 g·cm^–3^) are used,^34^ the values are between 112% and 158% of *q*_*R*_. Although there is uncertainty in the calculated *q*_*R*_ due to ambiguities in the choice of an appropriate surface tension value for these cold nanodrops, it appears that only nanodrops charged well above the Rayleigh limit undergo fission on the time scale of our measurements. This is almost certainly due to more highly charged nanodrops being more prone to fission than the less highly charged nanodrops shown in Figure 1C that only undergo water evaporation once in the electrostatic trap.

### Fast Fission

**Figure 2:**
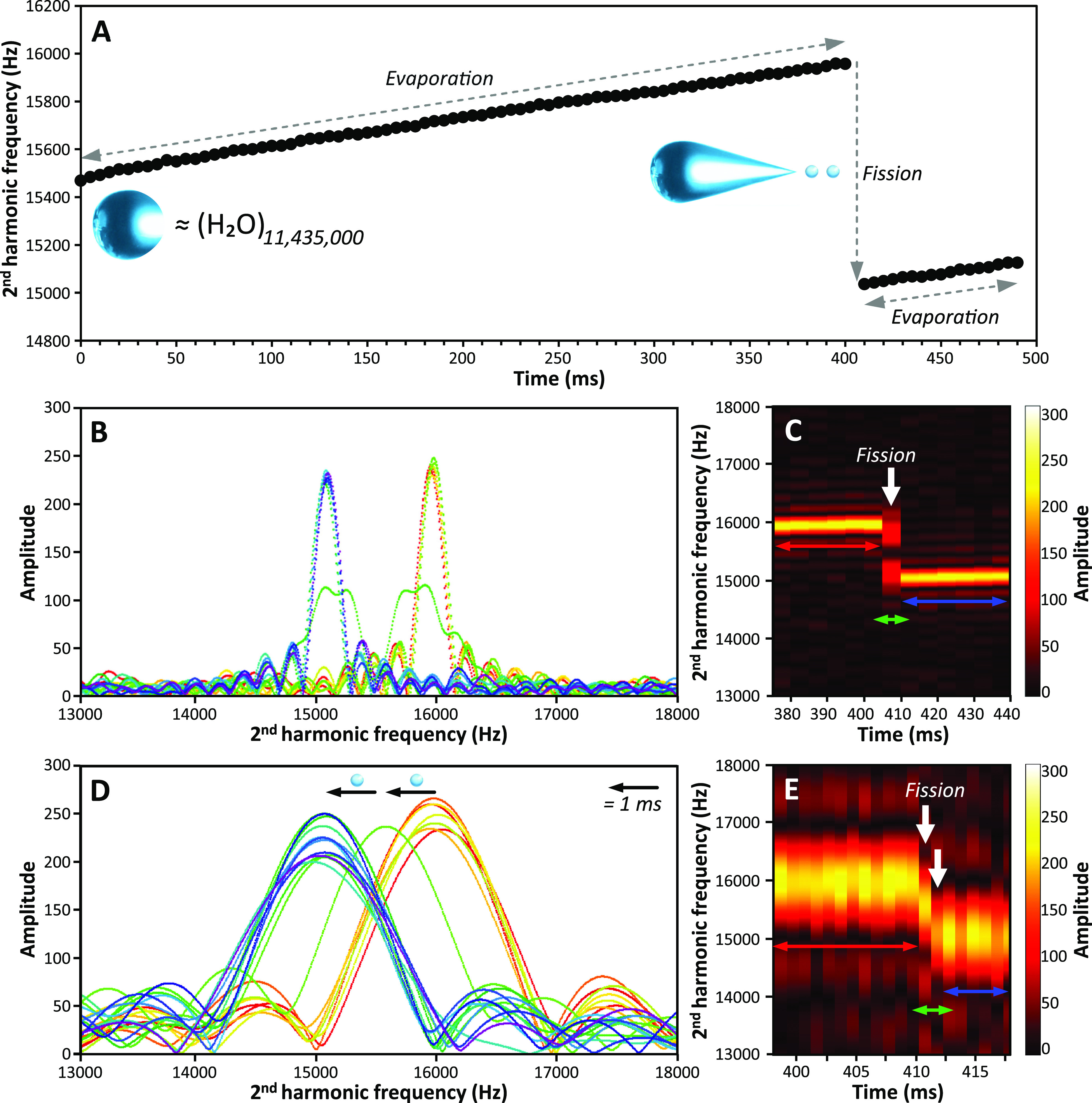
Frequency evolution of an 88 nm diameter charged water nanodrop trapped for 0.5 s in an electrostatic trap of a charge detection mass spectrometer. (A) Frequency evolution of an 88 nm diameter aqueous nanodrop for 0.5 s. (B–C) and (D–E) Time-resolved frequency analyses of this same nanodrop using a 5 ms and a 1 ms STFT segment length, respectively. The evolution of the frequency a few ms before (red-orange), after (blue-purple), and during (green) fission is shown in (B) and (D). Data in (C) and (E) are 2D maps of frequency and signal amplitude versus time in the fission region. White arrows in (C) and (E) indicate the fission event and the formation of progeny droplets.

The measured frequencies of an initial 88 nm water nanodrop (∼206 MDa; 1820 ± 13 charges) measured over 0.5 s is shown in Figure 2. The second harmonic frequency is shown for improved resolution, but both the second harmonic and the fundamental frequencies are used to determine *m*/*z* and *z*. The frequency steadily increases with time during the first 400 ms of trapping, then suddenly drops, and steadily increases again (Figure 2A). The evaporative mass lost from this nanodrop over the first 400 ms is ∼6 MDa, corresponding to a loss of ∼333,000 water molecules. At 400 ms, the water nanodrop mass is 200.4 ± 2.5 MDa and carries 1820 ± 13 charges corresponding to 159% of its Rayleigh limit charge. The sudden decrease in frequency results from fission that reduces the nanodrop mass to 198.2 ± 3.3 MDa and charge to 1685 ± 32 (150% of *q*_*R*_), corresponding to a loss of ∼7% charge (135 ± 34 charges) and ∼1% mass (2.1 ± 4.1 MDa). After fission, the nanodrop continues to lose mass from water evaporation and no additional fission occurs. The uncertainties in mass and charge are substantially higher than those determined for dry analyte ions^28,35^ because of the continuous mass change due to water evaporation and the limited observation time before and after the fission event.

Fission of water droplets that are charged above the Rayleigh limit has been reported previously and attributed to a kinetic effect.^28^ In addition, distortion of the nanodrop from a spherical geometry is necessary to produce progeny droplets. A spheroid-like shape, similar to the one reported by Duft et al. for the breakup of 48 μm diameter droplets of ethylene glycol,^13^ reduces Coulombic repulsion^36^ and therefore requires additional charges for spontaneous fission to occur. Solvent loss results in evaporative cooling that reduces the nanodrop temperature substantially, but this energy loss is balanced by energy deposition from collisions with background gas and by absorption of blackbody radiation.^37^ The steady loss of water in these experiments indicates that the nanodrops reach a steady-state internal effective temperature in the ion trap that is substantially below ambient temperature.^38−40^ The rate of water loss is similar before and after fission, indicating that the fission process itself does not significantly affect the internal effective temperature of the nanodrops. Although the nanodrop temperature is significantly lower than ambient, the structure of water in these nanodrops is not known. IR photodissociation spectroscopy on smaller ion-containing nanodrops (few nm diameters) trapped in vacuum for many seconds indicated that their surfaces are water-like but they can have crystalline ice-like cores.^41^ The extent to which these factors contribute to the observation that aqueous nanodrops that fission are charged >120% of the Rayleigh limit is unknown, but is worthy of further investigations.

Information about fission dynamics is obtained from short segment lengths in the short time Fourier transform (STFT) analysis. Time-resolved frequency data around the fission event at ∼405 ms using 5 and 1 ms STFT segment lengths are shown in Figure 2B,C and 2D,E, respectively. The nanodrop frequencies prior to fission (red-orange, 375–400 ms) and after fission (blue-purple, 410–440 ms) are readily distinguished from the associated side lobes^42^ (Figure 2B,C) and do not change significantly if shorter STFT segments are used (Figure 2D,E). However, there is a broad unresolved peak (green and white arrow in Figure 2B,C) in the 5 ms data at ∼405 ms, indicating that more than one fission event occurred. The dip in the center of this peak is due to interference that does not occur in the fundamental frequency (Supporting Information S1 and Figure S1). A shorter 1 ms segment length makes it possible to resolve an intermediate state (green peak in Figure 2D; white arrows in Figure 2E). These time-resolved data indicate that the fission event occurred within ∼2 ms through the formation of at least two progeny droplets (one at 409 ms and one at 410 ms; white arrows in Figure 2E). If the charge and mass lost during the fission event are evenly partitioned between the two progeny droplets, i.e., each progeny droplet is 1 MDa with 68 charges, they would be charged at ∼83% of the Rayleigh limit, consistent with some previously reported values for micron-size droplets.^43,44^

### Discrete Charge Emission Can Precede a Fission Event

**Figure 3:**
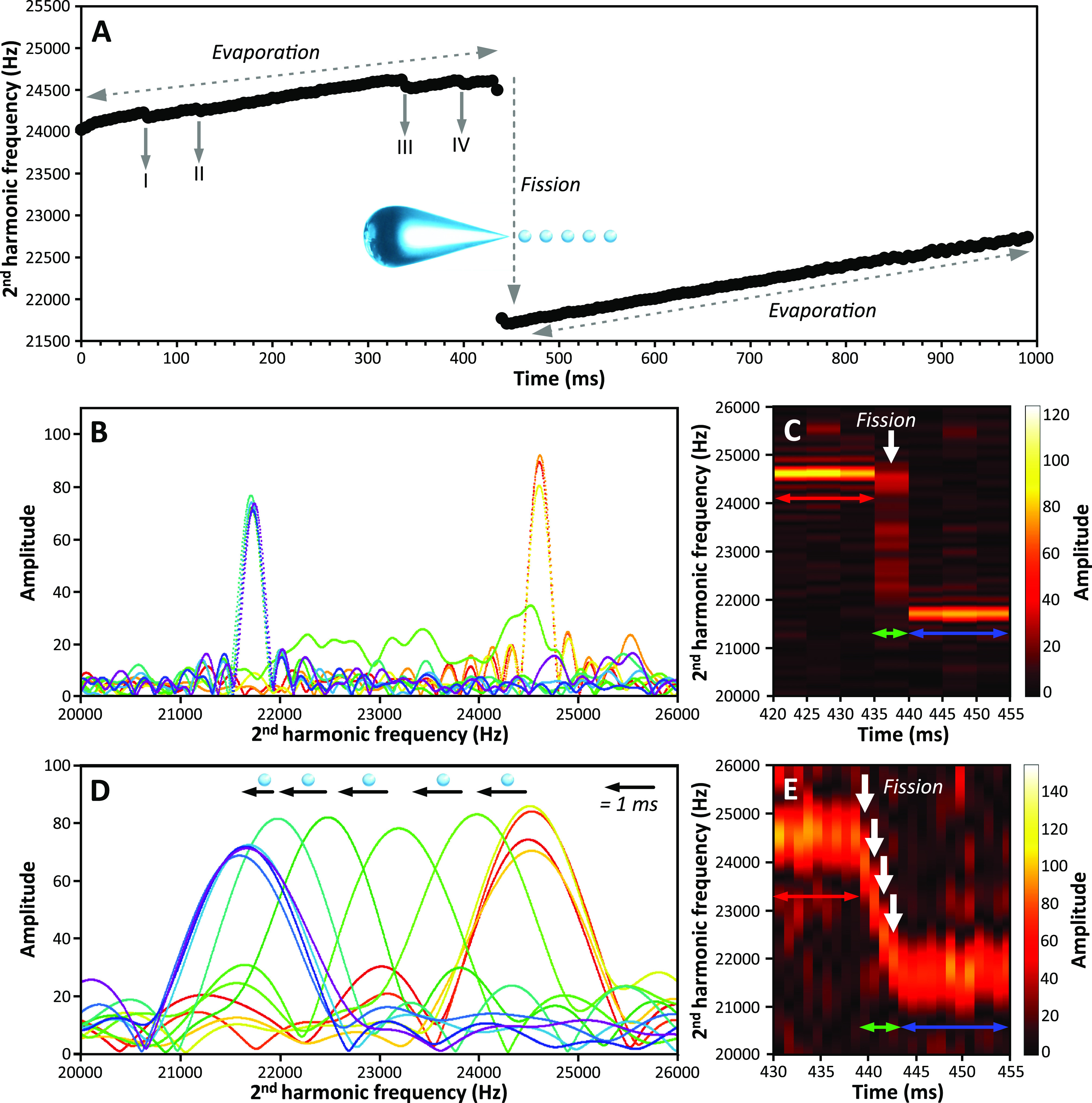
Frequency evolution of a 44 nm diameter charged water nanodrop trapped for 1 s in an electrostatic trap of a charge detection mass spectrometer. (A) Evolution of the nanodrop frequency with time. I, II, III, and IV indicate charge loss events prior to a large fission event at ∼430 ms. (B–C) and (D–E) Time-resolved frequency analyses using a 5 ms and a 1 ms STFT segment length, respectively. The evolution of the frequency a few ms before (red-orange), after (blue-purple), and during (green) fission is shown in (B) and (D). Data in (C) and (E) are 2D maps of frequency and signal amplitude versus time in the fission region. White arrows in (C) and (E) indicate the fission event and the formation of progeny droplets.

A similar sharp decrease in frequency occurs for a 26.8 ± 0.3 MDa (44 nm diameter) water nanodrop with 593 ± 6 charges (142% of *q*_*R*_, Figure 3). The frequency steadily increases between 0 and 430 ms due to solvent evaporation (Figure 3A), but there are also several discrete frequency drops (labeled I, II, III and IV in Figure 3A) that are consistent with small losses of *z* and *m*.^28^ The magnitude of these losses is too small to be accurately measured from the decrease in *z* and *m* due to the associated uncertainties. However, because these drops in frequency are small, neither the mass nor the energy of the nanodrop changes significantly during these events. Consequently, the variation of the squared frequency (Δ*f*^2^) can be directly related to the variation of charge (Δ*z*), which significantly improves the accuracy of the charge loss quantification. The charge losses calculated using this analysis at events I, II, III and IV are 3.3, 1.0, 5.7, and 1.7 charges, respectively (Figure 3A).

The large change in frequency at 430 ms is due to a large fission event, which reduces both the mass and charge of the nanodrop to 26.4 ± 0.3 MDa and 509 ± 6 charges. This corresponds to a charge loss of ∼14% (84 ± 8 charges) and a mass loss of ∼2% (0.4 ± 0.4 MDa). After fission, the nanodrop continues to evaporate with no additional charge loss. The time-resolved frequency data around the fission event at ∼430 ms are shown in Figure 3B,C, and 3D,E, for 5 and 1 ms STFT segment lengths, respectively. The nanodrop frequency immediately prior to (<430 ms, red-orange) and after (>440 ms, blue-purple) fission do not change significantly (Figure 3B–E). However, an intermediate broad and unresolved frequency (green, Figure 3B,C) is observed during the transition when a 5 ms STFT segment length is used, indicating multiple intermediate species with varying energy, *z*, and *m*. With a 1 ms STFT segment length, four intermediate frequencies can be distinguished (green peaks and white arrows in Figure 3D,E). These data indicate that the fission occurs by the production of at least five progeny nanodrops over the course of 5 ms. The progeny droplets are charged at ∼73% of the *q*_*R*_ if the charge and mass are equally partitioned.

### Fission Event Producing Many Progeny Droplets

**Figure 4:**
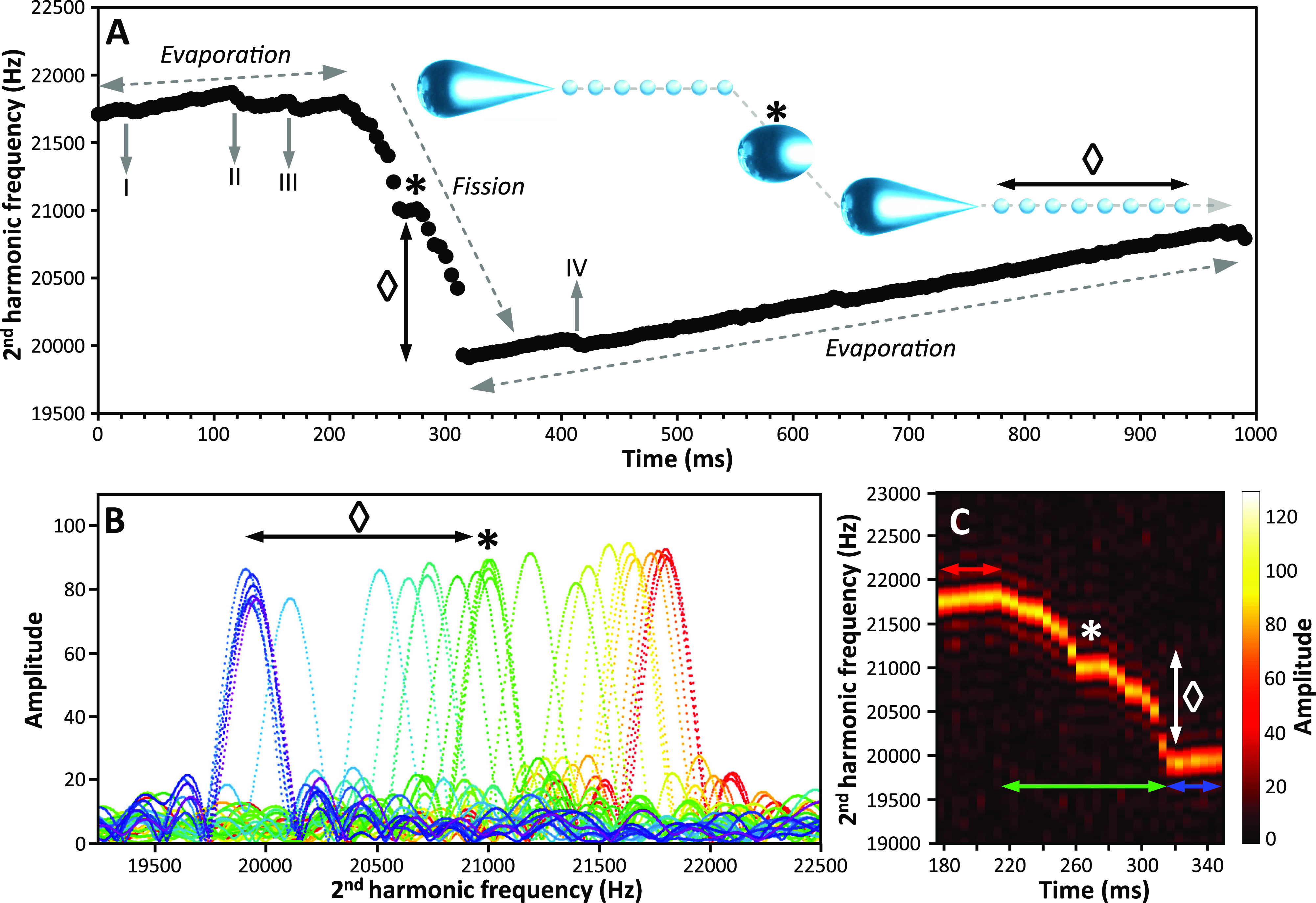
48 nm diameter charged water nanodrop trapped for 1 s in an electrostatic trap of a charge detection mass spectrometer. (A) Evolution of the nanodrop frequency as a function of time. I, II, III, and IV indicate small charge loss events prior to and after a large fission event. (B) and (C) Time-resolved frequency analyses using a 5 ms STFT segment length. The evolution of the frequency a few ms before (red-orange), after (blue-purple), and during (green) fission is shown in (B). Data in (C) is a 2D map of frequency and signal amplitude versus time in the fission region. The asterisks (*) highlight a stable intermediate nanodrop during the fission event. The diamond indicates a region of heterogeneous progeny droplets formation.

Discharge events can be more complex, as illustrated by the fission dynamics of a 35.6 ± 0.6 MDa water nanodrop (∼48 nm diameter) with 585 ± 7 charges (123% of *q*_*R*_, Figure 4). The nanodrop steadily evaporates during the first 215 ms while discrete charge loss events occur (labels I, II, III in Figure 4A), taking away approximately 1.0, 5.8, and 3.1 charges, respectively. The frequency then decreases rapidly between 215 and 315 ms due to a fission event (Figure 4A). The frequency does not change immediately prior to or after the fission (respectively red and blue peaks in Figure 4B,C; 5 ms STFT segment length). During this 100 ms-long fission event, there are a minimum of 15 discrete frequencies, each corresponding to the formation of at least one progeny droplet. Notably, the same frequency persists from 250 to 270 ms (four frequency peaks colored in green and labeled with an asterisk in Figure 4B,C), indicating that an intermediate nanodrop is stable for more than 20 ms before undergoing subsequent fission between 270 and 315 ms (highlighted by diamond in Figure 4B,C). The unequal frequency spacing between peaks indicates that this fission is not a continuous process and that the rate of charge emission varies over the course of the discharge. It also indicates that the progeny droplets have different sizes and charges. This is the first report of such heterogeneous fission behavior of water nanodrops.

After fission, the nanodrop has a mass of 34.3 ± 0.7 MDa and 535 ± 9 charges (112% of *q*_*R*_). For this fission, an overall charge loss of ∼8% (50 ± 11 charges) and mass loss of ∼4% (1.3 ± 0.9 MDa) occurred. Although it is not possible to decipher the individual characteristics of each progeny droplet, they would be charged at 14% of the Rayleigh limit if each of the 15 progeny were identical. From 315 ms to 1 s, the nanodrop continues to undergo evaporation, as well as an additional discrete charge emission of ∼2.3 charges (label IV, Figure 4A).

### Fission over a Long Time

**Figure 5:**
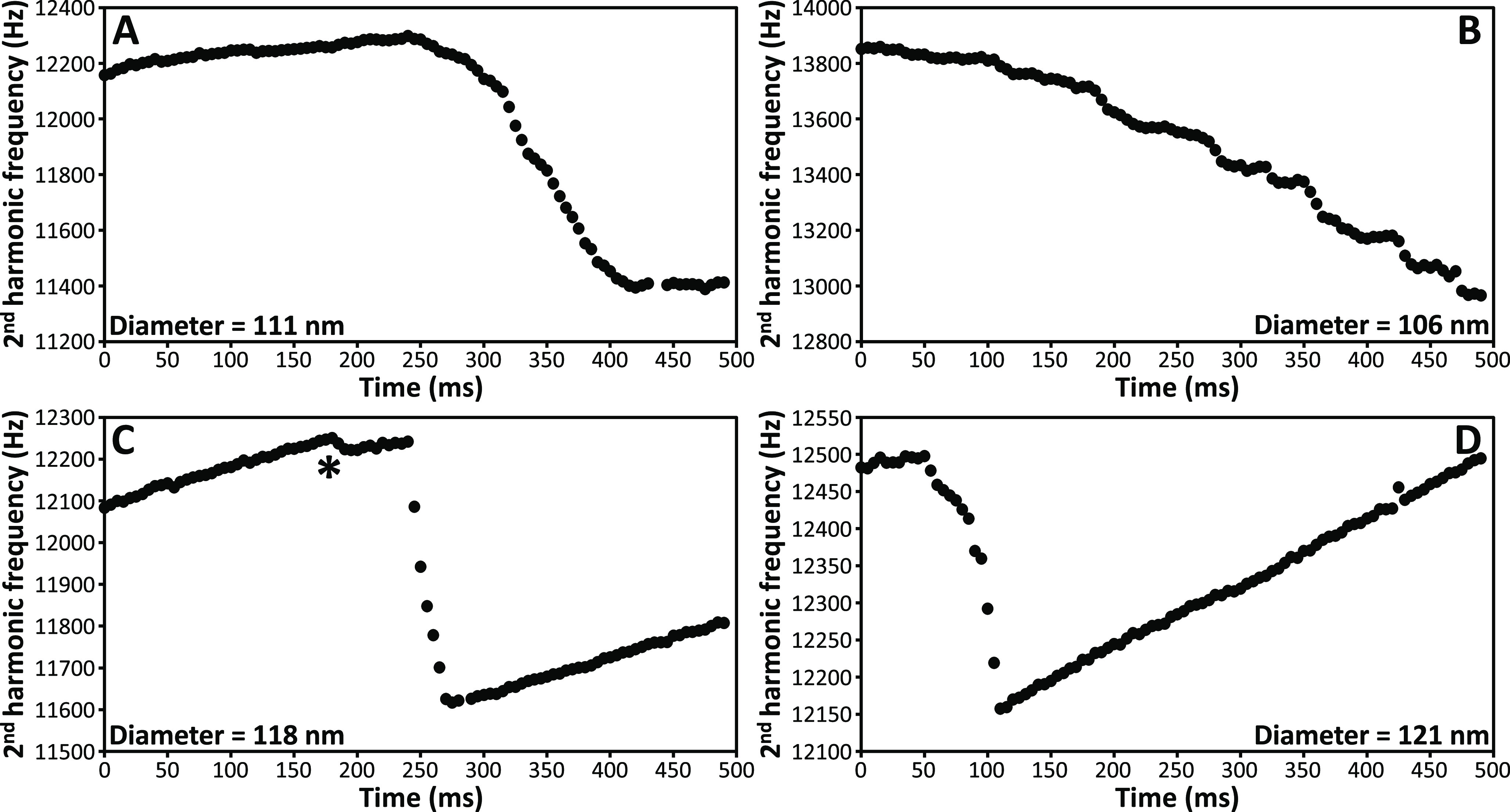
Time evolution of the frequency for four individual charged water nanodrops. (A) 111 nm diameter nanodrop (430.6 ± 5.4 MDa) with 2480 ± 22 charges prior to the fission. (B) 106 nm diameter nanodrop (373.0 ± 4.6 MDa) with 2563 ± 23 charges. (C) 118 nm diameter nanodrop (522.0 ± 5.7 MDa) with 2914 ± 25 charges. The asterisk (*) indicates fission with little charge and mass loss prior to a significantly larger fission event. (D) 121 nm diameter nanodrop (553.8 ± 8.4 MDa) with 3294 ± 49 charges.

Fission can occur by the nearly continuous formation of many progeny droplets. For example, the frequency of a 430.6 ± 5.4 MDa (111 nm diameter, Figure 5A) nanodrop with 2480 ± 22 charges (148% of *q*_*R*_) shows that fission lasts ∼175 ms and at least 30 progeny droplets are produced. The overall *z* and *m* losses during this event are ∼10% (238 ± 54 charges) and ∼3% (12.5 ± 9.2 MDa), respectively. Thus, each progeny droplet would be charged at ∼15% of *q*_*R*_ assuming that each of the droplets is identical.

Some nanodrops undergo fission throughout the entire 0.5 s trapping period. Figure 5B shows the frequency evolution of a 106 nm diameter nanodrop charged at 164% of the Rayleigh limit that undergoes stepwise reductions in frequency corresponding to sequential ejection of many small progeny droplets, each carrying relatively few charges. The total mass and charge losses are on the order of 9.0 ± 19 MDa and 251 ± 70 charges. These values have large uncertainties due to the continual change of the nanodrop kinetic energy as a result of water evaporation and fission.

### Rayleigh Fission: A Continuum of Pathways

Loss of small progeny droplets that carry away up to ∼5 charges often precede significant fission events where more charge is lost (Figure 5C, asterisk). Discharge events involving many progeny droplets can occur via a relatively even partitioning of charges between the droplets, or through an asymmetric distribution of the charge, affecting the rate at which the progeny droplets are produced. The frequency change between the sequential production of progeny droplets can be greater at the start of the discharge event than at its end, as occurs for a 118 nm nanodrop (522.0 ± 5.7 MDa charged at 158% of *q*_*R*_) shown in Figure 5C. This indicates that the progeny droplets carry away fewer charges as fission proceeds. This 118 nm nanodrop undergoes a total loss of 4.5% charge (132 ± 38 charges) and 3.7% mass (19.5 ± 8.6 MDa) during the 35 ms-long fission event. A similar size droplet (121 nm diameter charged at 172% of *q*_*R*_) shows the opposite behavior (Figure 5D). The progeny droplets produced at the beginning of the fission process carry away fewer charges than later progeny droplets.

In each of these examples, the progeny droplets were not detected, likely due to their relatively low charge and/or as a consequence of rapidly shifting frequencies due to evaporation of water molecules, resulting in signal below our detection limit. It is also possible that the progeny droplets formed have energy per charge values that are outside of the range of energies associated with stable trajectories within our electrostatic trap. We are pursuing an automated data analysis method that will make it possible to characterize a much larger number of fission events to obtain better statistics and to better search for progeny droplets that are formed.

## Conclusions

Fission of ∼100 nm charged aqueous nanodrops is significantly more heterogeneous than expected based on prior reports on the fission of larger micron-size droplets. The fissioning nanodrops investigated here are all charged well above the Rayleigh limit for a spherical droplet (between 123% and 172%) indicating that droplet distortion, likely into an elongated shape that reduces Coulomb repulsion, precedes progeny droplet formation. Although there are electric fields in the reflecting cones that make up the electrostatic ion trap, the associated field gradients are relatively low on the size scale of the nanodrops. Moreover, the nanodrops typically experience these fields thousands of times before fission and these events are uncorrelated with injection time, i.e., they are random. Combined, these results indicate that the fission events are spontaneous and not field induced. Spontaneous fission can occur rapidly, with loss of 100+ charges in 2 ms and production of only a few progeny droplets, or it can occur through the liberation of 30+ progeny droplets that carry away 200+ charges over 100+ ms. Fission reduces the nanodrop charge by between ∼4% and 14% and the mass lost ranges from <1% to 4%, consistent with previously reported data on micron-size charged droplets. The cold nanodrop temperature makes it possible to investigate the dynamics of these processes that have not been reported previously. These results show that nanodrop fission is a continuum of pathways encompassing a wide range of charge and mass losses that are distributed over a varying number of progeny droplets. These results, summarized in Table 1, also suggest that a charge loss mechanism commonly referred to as ion evaporation may not be a separate process to Rayleigh fission. Ion evaporation, as originally proposed by Iribarne and Thomson^45^ and incorporated in some mechanistic models of macromolecular charging in electrospray ionization,^46,47^ is based on preferential evaporation of small ions with more positive values of solvation free energy, i.e., many individual singly charged ions. In our experiments, a continuum of charge loss ranging from +1 to many dozens of charges occurs. The discrete loss of small progeny droplets that carry away little charge and mass occurs within the continuum of Rayleigh fission events that are observed here for 40–120 nm diameter aqueous nanodrops. Experiments aimed at determining how dissolved ionic species and other aqueous analytes may affect these fission processes are ongoing.

**Table 1:** Summary of the Fission Data on Positively Charged Pure Water Nanodrops

Properties *before* fission^a^	Properties *after* fission^a^	Charge loss (%)	Mass loss (%)	Minimum number of progeny droplets	progeny droplets *q*/*q*_*R*_^b^	Length of fission (ms)
86 nm	85 nm	7.4%	<1%	2^c^	83%	2 ms
200.4 ± 2.5 MDa	198.2 ± 3.3 MDa					
1820 ± 13 charges	1685 ± 32 charges					
*q*/*q*_*R*_ = 159%	*q*/*q*_*R*_ = 150%					
44 nm	44 nm	14.2%	1.5%	5^c^	73%	5 ms
26.8 ± 0.3 MDa	26.4 ± 0.3 MDa					
593 ± 6 charges	509 ± 6 charges					
*q*/*q*_*R*_ = 142%	*q*/*q*_*R*_ = 122%					
48 nm	48 nm	8.5%	3.6%	15^d^	14%	100 ms
35.6 ± 0.6 MDa	34.3 ± 0.7 MDa					
585 ± 7 charges	535 ± 9 charges					
*q*/*q*_*R*_ = 123%	*q*/*q*_*R*_ = 112%					
111 nm	110 nm	9.6%	2.9%	30^d^	15%	175 ms
430.6 ± 5.4 MDa	418.1 ± 7.5 MDa					
2480 ± 22 charges	2242 ± 49 charges					
*q*/*q*_*R*_ = 148%	*q*/*q*_*R*_ = 135%					
106 nm	105 nm	9.8%	2.3%	N.A.	N.A.	500 ms
373.0 ± 4.6 MDa	364.4 ± 18.4 MDa					
2563 ± 23 charges	2312 ± 66 charges					
*q*/*q*_*R*_ = 164%	*q*/*q*_*R*_ = 150%					
118 nm	117 nm	4.5%	3.7%	7^d^	14%	35 ms
522.0 ± 5.7 MDa	502.5 ± 6.5 MDa					
2914 ± 25 charges	2782 ± 28 charges					
*q*/*q*_*R*_ = 158%	*q*/*q*_*R*_ = 153%					
121 nm	120 nm	3.7%	1.9%	12^d^	13%	60 ms
553.8 ± 8.4 MDa	543.3 ± 5.4 MDa					
3294 ± 49 charges	3171 ± 14 charges					
*q*/*q*_*R*_ = 172%	*q*/*q*_*R*_ = 168%					

a Values are the nanodrop diameter, mass, the charge, and charge relative to the Rayleigh limit (*q*/*q*_*R*_), respectively. *q*/*q*_*R*_ is calculated using the surface tension and the density of water at 0 °C in eq 1.

b The charge of the progeny droplet relative to the Rayleigh limit (*q*/*q*_*R*_) calculated assuming an equipartition of charge and mass for each progeny droplet. *q*/*q*_*R*_ is calculated using the surface tension and the density of water at 0 °C in eq 1.

c Determined from 1 ms STFT segment length.

d Determined from 5 ms STFT segment length.

## Methods

### Charge Detection Mass Spectrometry Measurements

Experiments were performed using a new electrostatic ion trap-based charge detection mass spectrometer that does not use energy selective ion optics. A complete description of this instrument and operating parameters is given elsewhere.^35^ In brief, the instrument is composed of five distinct regions: an electrospray ionization (ESI) source, a heated capillary and ion funnel region, a quadrupole thermalization and accumulation region (composed of three successive quadrupoles), an acceleration region, and finally an electrostatic ion trap where the current induced by individual charged nanodrops oscillating within the cone electrodes is measured and used to determine their charge (*z*), mass-to-charge ratio (*m*/*z*), and mass (*m*). Radio frequencies for the ion funnel and quadrupoles were optimized for efficient transmission of the charged nanodrops. Frequencies of 200 kHz, 100 kHz, 65 kHz, and 65 kHz, respectively, were used for the ion funnel and for the three consecutive quadrupoles. The pressure in the final electrostatic ion trap region was ∼1 × 10^–8^ Torr, and ions were trapped and measured for a period of either 0.5 or 1.0 s. The instrument was operated at room temperature.

### Production of Charged Aqueous Nanodrops

Aqueous nanodrops were produced from purified water using electrospray ionization (positive ionization) via a HESI-II Probe (ThermoFisher Scientific, San Jose, CA) adapted to the charge detection mass spectrometry (CDMS) instrument inlet. The inner diameter of the stainless-steel ESI emitter was 0.1 mm. The emitter was oriented at 45° to the instrument axis and was positioned ∼1.5 cm from the instrument inlet capillary. A positive electrospray voltage of 4.0–4.7 kV was applied to the emitter via an external high voltage power supply (Bertan Associates Inc., Model 315B, Hicksville, NY). Deionized water, purified to a resistivity of 18.2 MΩ·cm (at 25 °C) using a Milli-Q Gradient ultrapure water purification system (Millipore, Billerica, MA), was introduced to the ESI source at a flow rate of 600 μL per hour using a 0.250 mL gastight Hamilton syringe coupled to a syringe pump (Harvard Apparatus, Model 22, South Natick, MA). No unexpected safety hazards were encountered. To reduce evaporation of water molecules from the nanodrops in the early stage of the instrument, the temperature of the heated capillary at the entrance of the instrument was set to 80 °C, a value that is lower than the normal operating temperature, typically between 120 and 140 °C.

### Production of Charged 100 nm Diameter Polystyrene Nanospheres

Polystyrene nanospheres with diameters of 101 ± 3 nm were obtained from Thermo Scientific (catalog no. 3100/3100A). The sample was diluted by a factor of 500 in 0.5% aqueous acetic acid. Charged nanospheres were produced using a nanoESI ionization source. A borosilicate capillary pulled to a tip with an inner diameter of 5–6 μm was filled with the nanosphere-containing solution and placed ∼5 mm from the instrument inlet. A voltage between +1.3 and +1.7 kV was applied to a platinum wire in contact with the solution to initiate electrospray. The heated capillary at the entrance of the instrument was 140 °C. The nanospheres were trapped for 1 s and analyzed using a STFT segment length of 50 ms and a 5 ms overlapping step. Additional instrumental details and a more in depth description of the analysis are given elsewhere.^35^

### Standard Analysis of CDMS Data

Standard analysis of CDMS data is described extensively elsewhere.^30−32,35^ As a charged nanodrop passes through the detector cylinder embedded within the electrostatic trap, it induces a current that is converted into a voltage signal by a charge-sensitive preamplifier (Amptek A250 CoolFET, Bedford, MA) inside the vacuum chamber where the electrostatic trap is located. The pulse amplitudes of this signal are proportional to the charge of the nanodrop. The signal is then directed to a custom bandpass filter (passband from ∼1 kHz to ∼300 kHz) located outside of the vacuum chamber. The signal is digitized at 1 MHz (AlazarTech ATS9120, Pointe-Claire, QC, Canada) and further analyzed using a custom Python program.^32^

Peaks corresponding to individual nanodrop signals within the 0.5 or 1 s measured transients were traced and fit using short-time Fourier transform (STFT)-based methods described in detail elsewhere.^32,42^ Nonoverlapping segments of 5 ms were used to step across the time-domain data and track the evolution of the nanodrops frequencies and amplitudes as a function of time. The individually traced nanodrop frequencies together with the amplitudes of the first and second harmonics were then used to calculate segment-by-segment values for nanodrop energy per charge, charge, mass-to-charge ratio, and mass.^30−32,48,49^

Calibration values used to convert frequency to mass-to-charge ratio and convert harmonic amplitude ratios (HARs) to nanodrop energies were determined from SIMION simulations.^30,31,35^ Calibration values used to convert fundamental amplitude to charge were determined using standards of known mass and a trap-specific SIMION simulation-based correction of raw amplitudes based on nanodrop energies.^49^

Except where otherwise stated, the mass and charge of the aqueous nanodrops were determined using this STFT method. Because substantial water evaporation occurs during the trapping period, the mass of a nanodrop before and after a fission event corresponds to average values that were determined from the same length of time, i.e., the same number of data points, prior to and after a fission event. This time is different for each individual aqueous nanodrop, with values ranging between 50 ms (= 11 data points) and 430 ms (= 87 data points) and depends on when the fission event occurred within the trap. The mass loss is obtained from the difference in mass before and after the fission event. The charge reported for the nanodrops before a fission event corresponds to an average value of the individual charge values calculated over the entire period that precedes fission. The same strategy, using the entire period after fission, was used to determine the charge of the nanodrops after a fission event. The charge loss is then calculated from the difference in charge before and after the transition. Effects of minor emission events corresponding to the loss of one or just a few charges that occurred before and/or after the main fission event were neglected. These charge emission events only release a very small number of charges from the nanodrop surface in comparison to the charge lost in larger fission events and therefore represent a negligible proportion of its total charge. It is important to note that in the case of the nanodrop reported in Figure 2, error on the charge after the transition is larger than before the fission because fewer data points were included in the calculation of the mean charge state and its associated error. 81 individual data points were used to calculate the mean before the fission event, while only 15 individual data points were available to calculate these quantities after fission. The latter case approaches the sampling limit required to produce a Gaussian distribution that is representative of the true distribution and thus results in a larger error in the estimation of the mean. These statistical consequences also applied to the nanodrop presented in Figure 5D. Ten and 67 individual data points were used to calculate the average charge state and the error before and after fission, respectively.

### Determination of Water Evaporation from Energy and Frequency Changes

Aqueous nanodrops steadily undergo water evaporation during the trapping period so that their mass is steadily and substantially decreasing throughout the trapping time. This change in mass leads to a concomitant decrease of its total energy per charge that manifests as a steady increase in the nanodrop frequency with time. Both the energy and the frequency change rapidly over the lifetime of the nanodrops, and the use of very short time segments necessary to obtain steady frequency and accurate amplitude values makes it difficult to measure the change in energy with high precision. Because energy cannot be measured with high precision, it further limits our ability to accurately evaluate the extent of mass change during the slow evaporative process. Because the mass loss does not depend on the position of the nanodrop within the electrostatic trap, the change in the average percent energy loss is proportional to the average percent mass loss according to the relationship shown in eq 3:^31^
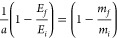
3where *E*_*i*_ and *m*_*i*_ are, respectively, the total energy per charge and mass of the nanodrop as it enters the analyzer, *E*_*f*_ and *m*_*f*_ are, respectively, the total energy per charge and mass of the nanodrop at the end of the evaporation period, and *a* is a proportionality constant determined from SIMION simulations that represents the average kinetic energy of nanodrops relative to their total energy during the trapping period. For the ion trap used here, *a* = 0.5018, meaning that the nanodrops have, on average, ∼50% of their total energy as kinetic energy. Because mass losses carry away only kinetic energy, a mass loss of 1% of the total mass results in ∼0.5% decrease in the total energy per charge of the nanodrop. From eq 3, we can express the final mass of the nanodrop as a function of its total energy per charge (eq 4):
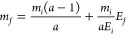
4

The change of the nanodrop frequency (*f*) is linked to the change in total energy per charge through the energy dependent value *C*(*E*) according to eq 5, where *z* is the charge state:
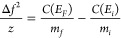
5The expression of *C*(*E*) is described elsewhere and is a function of the total energy per charge (*E*) of the nanodrop.^48^

By substituting eq 4 into eq 5, the total energy per charge (*E*_*f*_) of the nanodrop at the end of the evaporation period can be expressed as a polynomial equation. To solve this equation, we use the properties of the initial nanodrop (the charge state *z*, the initial total energy per charge *E*_*i*_ and the initial mass *m*_*i*_) determined from the standard analysis procedure. In the case of the initial 88 nm diameter nanodrop shown in Figure 2, the charge state *z*, the initial total energy per charge *E*_*i*_ and the initial mass *m*_*i*_ were defined as average values and obtained from the first 400 ms of evaporation. As such, *z* = 1820 charges, *E*_*i*_ = 244.3 eV/charge and *m*_*i*_ = 198.9 MDa. The roots then provide a real solution for *E*_*f*_ = 240.7 eV/charge. Substituting *E*_*f*_ into eq 3 leads to a loss of energy of 1.48% during the 400 ms of evaporation, which corresponds to a mass loss of 2.95%.

From the standard analysis procedure, we determined that the initial nanodrop mass was ∼206 MDa. The present analysis suggests that ∼6 MDa of water molecules (∼333,000 water molecules) are lost via evaporation over the course of a 400 ms trapping period.

### Short Time Fourier Transform Analysis

The frequency of motion and the signal amplitude of a nanodrop are obtained from short time Fourier transform analysis (STFT). In this STFT analysis, the time-domain data or transient is segmented into smaller transients with nonoverlapping windows of either 5 or 1 ms. A discrete Fourier transform is performed on each segment, and the frequency and the amplitude of the nanodrop during each short time period are obtained. In our analysis, the first 5 ms of a transient, which contains large pulses resulting from the opening and closure of the electrostatic trap, is discarded, and the transient is segmented starting from this point (Figure S2A,B). With these analysis parameters, we observed an STFT segment with meaningful amplitudes spread across a range of frequencies, resulting in a broad and unresolved signal (Figure S2, green). This effect indicates that the frequency of motion varied significantly over the time period of that particular STFT segment.

To better bracket the signal during the fission event, we modified the starting point of the STFT analysis of the transient, i.e., we added a phase shift. Figure S2C–H shows the effect of adding different extents of phase shifts in the STFT analysis. The frequency of the nanodrop before (red) and after (blue) the transition do not vary significantly over the corresponding 5 ms STFT segments and are therefore not significantly affected by the phase shift. However, the observed frequencies of the nanodrop during the transition (green) are affected by the phase shift, because time periods where the frequency change occurs are partitioned differently across the STFT segments. Figure S2G,H displays the result of the STFT analysis performed with a phase shift of 3 ms relative to the initial analysis in Figure S2A,B. The frequency measured during the transition is still unresolved and broad but is more evenly spread over the STFT segment where the frequency transition occurs. These data were further processed with a phase shift of 3 ms because it provides the clearest delineation of the fission event. If necessary, the phase shift was adapted for each nanodrop in order to evenly bracket the changing signal during the fission events.

### Determination of Charge Loss from Small-Scale Frequency Drops

In addition to the large changes in frequency that correspond to fission events, much smaller changes in frequency, associated with what were previously called charge emissions, also occur (Figure 3).^28,50^ These small changes in frequency correspond to a negligible mass loss and a minimal charge loss compared to the larger fission events. As a consequence, it is challenging to obtain a mass loss and a charge loss for these small frequency changes according to the standard analysis procedure because of the relatively large uncertainty associated with subtracting large charge and mass numbers. To obtain more quantitative information on these small-scale events, we made the approximation that the total mass and total energy per charge of the nanodrop remained unchanged after the emission events and that the charge loss is very small. As such, the relationship between the number of charges Δ*z* released during the emission event and the corresponding drop in frequency Δ*f*^2^ can be written as (eq 6):
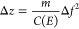
6where *m* and *C*(*E*) are, respectively, the total mass of the nanodrop and the energy-dependent constant, both averaged over the time segment of interest. The evolution of the nanodrop second harmonic frequency as a function of time was fit with a linear equation for the time segments before and after the charge emission. The resulting equations were used to accurately calculate the second harmonic frequency of the nanodrop just before and after this event. Using the second harmonic frequency allowed us to measure the frequency difference more precisely. Based on the known properties of Fourier series expansion of a square pulse in the frequency domain, the second harmonic frequency equals two times the fundamental frequency and was thus used to calculate the fundamental frequency of the nanodrop before and after the charge emission, *f*_*before*_ and *f*_*after*_ respectively. Δ*f*^*2*^ was then calculated from the so-determined fundamental frequency (eq 7):
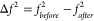
7

## Data Availability

All data, experimental procedures, and data analysis methods are available in the main text or the Supporting Information.

## Supporting Information Available

Interpretation of signal interference in the second harmonic frequency, additional details on the effect of phase shifts in short time Fourier transform analysis (PDF)
Raw data S1–S7 (XLSX)
