# Supplementary material for: Dynamics of Rayleigh Fission Processes in ∼100 nm Charged Aqueous Nanodrops
Source: ACS Cent Sci. 2023 May 31;9(8):1611–22. doi: 10.1021/acscentsci.3c00323 (PMC10451037; doi:10.1021/acscentsci.3c00323)
Supplement: Supplementary file 1 — oc3c00323_si_001.pdf [file oc3c00323_si_001.pdf]

Supporting Information for

Dynamics of Rayleigh Fission Processes in ~100 nm Charged Aqueous Nanodrops

Emeline Hanozin, Conner C. Harper, Matthew S. McPartlan and Evan R. Williams*

Department of Chemistry, University of California; Berkeley, CA, 94720, USA.

*Corresponding author: erw@berkeley.edu

The PDF file includes:

S1. Interference in the second harmonic frequency.

Figure S1. Time-resolved frequency analysis based on a segment length of 5 ms. Comparison of the fundamental and second harmonic frequencies for an 88 nm diameter water droplet.

Figure S2. Effect of phase shifts in Short Time Fourier Transform analysis.

Other Supplementary Materials for this manuscript include the following:

Excel sheet: Raw data S1 to S7:

S1. Nanodrop #1 – 86 nm diameter – evolution of the fundamental and harmonic frequencies with time.

S2. Nanodrop #2 – 44 nm diameter – evolution of the fundamental and harmonic frequencies with time.

S3. Nanodrop #3 – 48 nm diameter – evolution of the fundamental and harmonic frequencies with time.

S4. Nanodrop #4 – 111 nm diameter – evolution of the fundamental and harmonic frequencies with time.

S5. Nanodrop #5 – 106 nm diameter – evolution of the fundamental and harmonic frequencies with time.

S6. Nanodrop #6 – 118 nm diameter – evolution of the fundamental and harmonic frequencies with time.

S7. Nanodrop #7 – 86 nm diameter – evolution of the fundamental and harmonic frequencies with time.

S1. <u>Interference in the second harmonic frequency.</u>

Figure 1D in the main text shows a destructive interference for the broad and unresolved second harmonic frequency corresponding to the intermediate state of the nanodrop (green). This destructive interference is an artifact of observing the second harmonic frequency that occurs due to the frequency spacing of the initial and final frequency peaks. As shown in Figure S2, this interference does not occur for the fundamental frequency peaks due to the smaller frequency spacing between peaks and is therefore not associated with a specific behavior in the nanodrop motion during the trapping period.

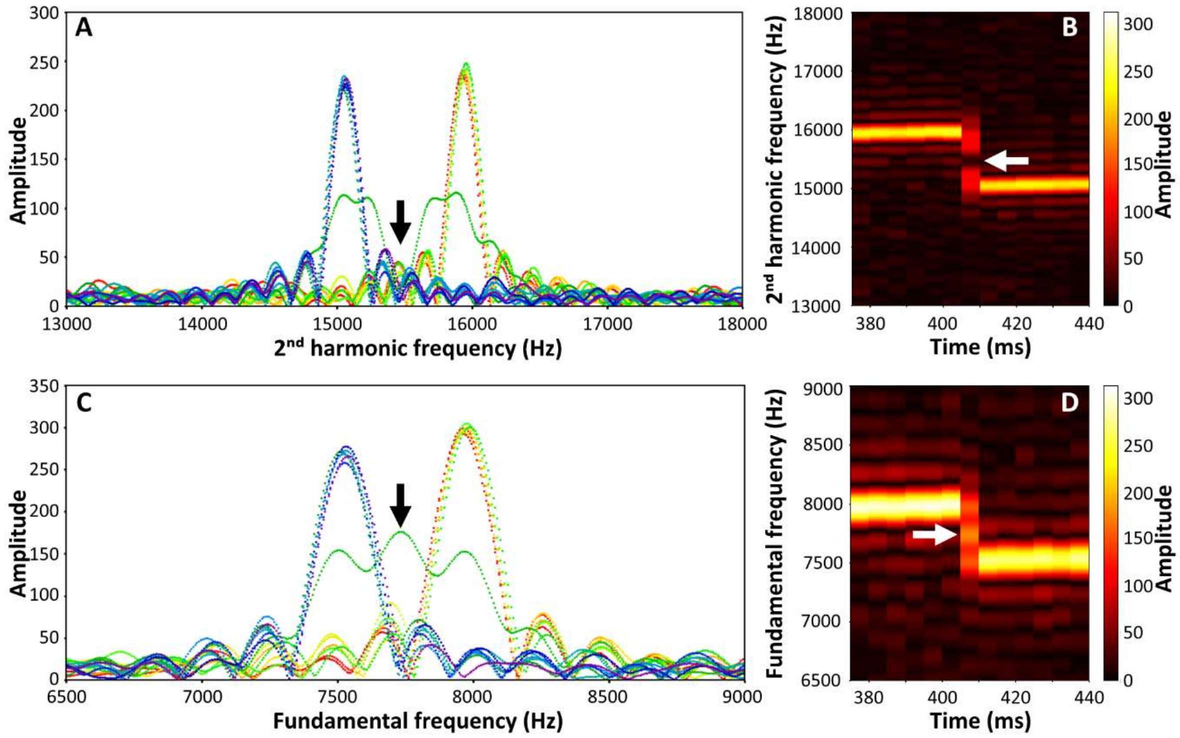

Figure S1. Time-resolved frequency analysis based on a segment length of 5 ms. (A and B) Second harmonic frequency. (C and D) Fundamental harmonic frequency. The evolution of the frequency a few ms before and after the transition as well as during the transition is shown in (A) and (C), while (B) and (D) provide 2D maps of the transition region as a function of the trapping time.

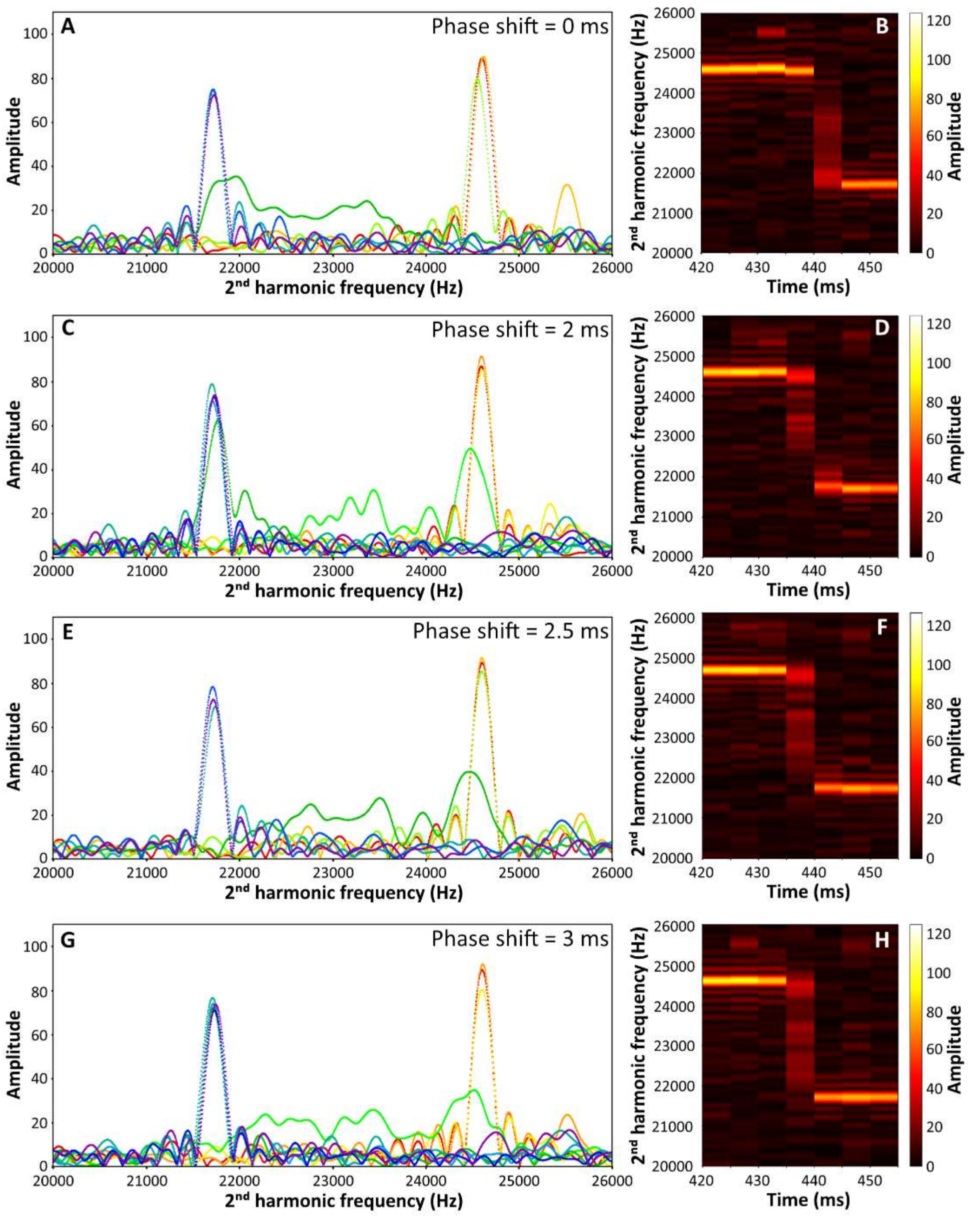

Figure S2. Effect of phase shifts in Short Time Fourier Transform analysis. Time-resolved frequency analysis based on a segment length of 5 ms. Data are shown for the second harmonic frequency of the nanodrop presented in Figure 2 of the main text. STFT analysis are performed with different phase shifts: 5 ms (A and B), 7 ms (C and D), 7.5 ms (E and F) and 8 ms (G and H). The evolution of the frequency a few milliseconds before (red) and after (blue) the transition as well as during (green) the transition is shown in the left panels, while the right panels provide 2D maps of the transition region as a function of the trapping time.
